# Prodromal Cognitive Deficits and the Risk of Subsequent Parkinson’s Disease

**DOI:** 10.3390/brainsci12020199

**Published:** 2022-01-31

**Authors:** Daniël H. B. Speelberg, Jules M. Janssen Daalen, Bastiaan R. Bloem, Jean-François Gagnon, Bart Post, Sirwan K. L. Darweesh

**Affiliations:** 1 Department of Neurology, Center of Expertise for Parkinson & Movement Disorders, Donders Institute for Brain, Cognition and Behaviour, Radboud University Medical Center Nijmegen, 6525 GC Nijmegen, The Netherlands; daniel.speelberg@radboudumc.nl (D.H.B.S.); jules.m.janssendaalen@radboudumc.nl (J.M.J.D.); bas.bloem@radboudumc.nl (B.R.B.); bart.post@radboudumc.nl (B.P.); 2 Department of Psychology, Université du Québec à Montréal, Montreal, QC H2X 3P2, Canada; gagnon.jean-francois.2@uqam.ca

**Keywords:** Parkinson’s disease, systematic review, meta-analysis, prodromal, risk assessment, longitudinal studies, cognitive deficits, cognitive domains, executive dysfunction, memory, attention

## Abstract

Background: There is growing interest in identifying individuals who are in the prodromal phase of Parkinson’s disease (PD), as these individuals are potentially suitable for inclusion in intervention trials to prevent clinically manifest PD. However, it is less clear whether—and to what extent—cognitive deficits are present in prodromal PD. Methods: A systematic query was conducted through PubMed and Embase for prospective observational cohort studies that (a) assessed cognitive performance in individuals free of manifest PD at baseline and (b) subsequently followed up participants for incident PD. We grouped the results by cognitive domain, and for domains that had been reported in at least three separate studies, we performed random-effects, inverse variance meta-analyses based on summary statistics. Results: We identified nine articles suitable for inclusion, with a total of 215 patients with phenoconversion and 13,524 individuals remaining disease-free at follow-up. The studies were highly heterogeneous in study design, study population, and cognitive test batteries. Studies that included only cognitive screening measures such as MMSE or MoCA reported no association between worse cognitive performance and onset of manifest PD (combined odds ratio 1.08; 95% confidence interval 0.66–1.77). By contrast, studies that used extensive cognitive testing batteries found that global cognitive deficits were associated with an increased risk of manifest PD. In domain-specific analyses, there was evidence for an association between worse executive functioning (OR 1.45; 95% CI 1.10–1.92), but not memory (OR 1.20; 95% CI 0.85–1.70) or attention (OR 0.98; 95% CI 0.23–4.26), and clinically manifest PD. Conclusion: Although some caution due to high heterogeneity among published studies is warranted, the available evidence suggests that global and executive cognitive deficits are prodromal features of PD. Collaborative prospective studies with extensive cognitive test batteries are required to shed light on domain-specific deficits, temporal relations, and subgroup differences in prodromal cognitive deficits in PD.

## Key Points:

- Published studies on cognitive performance in prodromal Parkinson’s Disease (PD) have been highly heterogeneous in study design, study population, and cognitive test batteries.
- Studies that included only cognitive screening measures such as MMSE or MoCA reported no association between cognitive performance and onset of manifest PD.
- By contrast, studies that used extensive cognitive test batteries found that global and executive cognitive deficits were associated with an increased risk of manifest PD.
- In domain-specific analysis, executive dysfunction was significantly associated with conversion to manifest PD.
- Collaborative prospective studies with extensive cognitive test batteries are required to shed light on domain-specific deficits, temporal relations, and subgroup differences in prodromal cognitive deficits in PD.

## 1. Introduction

Parkinson’s disease (PD) is a neurodegenerative disorder characterized primarily by degeneration of dopaminergic neurons. Current criteria for diagnosis include the motor symptoms bradykinesia with either rest tremor, rigidity, or both combined with supportive criteria and the absence of exclusion criteria [1]. However, it has been estimated that these symptoms become manifest only when at least 60% of nigrostriatal dopaminergic neurons have already been degenerated [2]. Therefore, the concept of a prodromal phase in PD, i.e., a phase during which degeneration of neurons has started, but the classic motor symptoms are not yet apparent, was introduced [3]. Besides the hallmark locations of the striatum and the substantia nigra, other brain locations are known to experience degeneration in early stage [4,5]. During the prodromal disease phase, nonmotor symptoms such as hyposmia, rapid eye movement sleep behaviour disorder (RBD), and autonomic dysfunction (such as orthostatic hypotension) gradually arise [6,7]. Timely recognition of this prodromal phase might provide an early opportunity for intervening in the underlying pathological processes, with the aim of postponing or even preventing the onset of clinically manifest PD, which is the phase that starts when a clinical diagnosis is made based on overt symptomatology [8]. In addition, genetic risk factors (such as LRRK2 and GBA, for example) have been established and may aid in identifying individuals in their prodromal phase [9,10]. 

Defining this prodromal phase is the subject of much ongoing research. Presence of cognitive deficits is well established as a feature of later stages of PD and can even occur in patients shortly after their diagnosis. It has recently become clear that mild cognitive deficits in several domains, such as executive function, are also already manifest in the prodromal phase [11], and thus, cognitive deficits were recently implemented in the updated Movement Disorders Society (MDS) research criteria for prodromal PD [12]. However, there is a relative lack of insight as to which cognitive domains are affected in prodromal PD.

In this systematic review, we present the results of longitudinal cohort studies on cognitive impairment in prodromal PD with the aim to quantitate the association between domain-specific cognitive deficits and the subsequent onset of manifest PD. 

## 2. Methods

This systematic review was performed in accordance with the Preferred Reporting Items for Systematic Reviews and Meta-Analyses (PRISMA) statement and the Cochrane Handbook for Systematic Reviews of Interventions.

### 2.1. Search Strategy

The search strategy (as presented in the Supplementary Materials) was performed in PubMed and Embase in April 2021. Studies were selected by determining relevance using title and abstract by two researchers (D.H.B.S., J.M.J.). Nonoriginal investigations (such as reviews) were checked for potentially relevant references. References of all included articles were searched for additional eligible studies. This process did not lead to the additional inclusion of articles. 

### 2.2. Selection Criteria

All peer-reviewed articles and prospective or nested case-control studies were considered. We included both population-based cohort studies and enriched cohort studies. Enriched cohort studies were defined as cohorts in which selection of individuals was based on a high-risk trait of conversion to manifest PD, e.g., individuals with idiopathic RBD or hyposmia, first-degree relatives of PD patients, or carriers of an *LRRK2* or *GBA* mutation. We required that a study assessed global cognition or domain-specific cognitive function using validated tests at baseline as part of the study protocol. We excluded studies in patients with pre-existent neurodegenerative diseases (including but not limited to dementia and parkinsonism) and studies presenting disease-converting results without further detailing the PD subgroup-specific outcome. The main outcome was incident PD. We required all articles to report at least 5 incident PD cases in order to reduce the risk of inflation of odds ratios. One study did not meet this requirement and was subsequently excluded [13]. We meta-analysed cognitive scores if estimates were available and interpretable from at least three different cohorts. 

### 2.3. Data Analysis

The cognitive tests employed in the studies were analysed by domain. Recognizing that each cognitive test may span multiple cognitive domains, we used literature that was recent and of sufficient size to determine a validated consensus to allocate cognitive tests to their respective strongest correlating domains [14,15,16,17]. An overview of the resultant allocation of cognitive domains is presented in Supplementary Table S1. This led to allocations for tests in the PARS and Amsterdam study that in some specific instances differed from the original allocation in the study. For every conflicting study and allocation decision, we describe this process in detail in the Supplementary Materials. 

If necessary, odds ratios presented in this article were converted so that an odds ratio greater than 1 indicated a higher risk of conversion to clinical PD for worse cognitive test performance. Throughout this article, we refer to studies based on the cities or cohorts they were conducted in for readability.

If risk estimates were unavailable, the corresponding authors of the included articles were contacted to provide them to us. Data on z-scores per test for one included article, the Montreal cohort [18], were made accessible by the corresponding author. Additionally, data on baseline Teng-MMSE scores in the ABC study [19] were made accessible and converted. We could not include studies in the meta-analyses that presented only other risk metrics (e.g., median and *p*-value [20,21]). 

Outcomes were converted to odds ratios per standard deviation scoring worse on their respective cognitive tests for comparison using Hedge’s G [22]. Cognitive domains for which three or more studies reported comparable risk metrics were analysed. Consequently, a random-effects inverse variance analysis was performed in the domains of global cognition, executive function, learning and memory, and attention. To leverage data from multiple cognitive tests on a single cognitive domain, a composite score was calculated for executive function in the Rotterdam [23] and Montreal [18] cohorts, for learning and memory in the Rotterdam cohort [23], and for attention in the Montreal cohort [18]. The Hartung–Knapp method was used to adjust for heterogeneity between studies and the low number of studies per analysis [24].

## 3. Results

### 3.1. Studies Included

**Figure 1:**
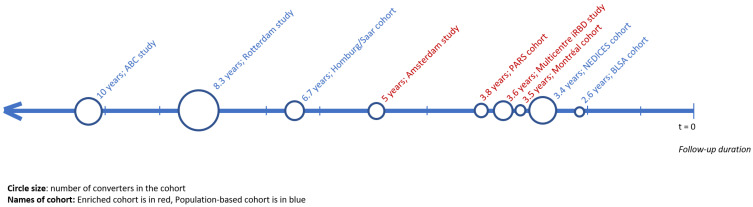
Timeline of the mean or median follow-up duration of all included studies with additional depiction of study size and nature of cohort (population-based or enriched).

**Figure 2:**
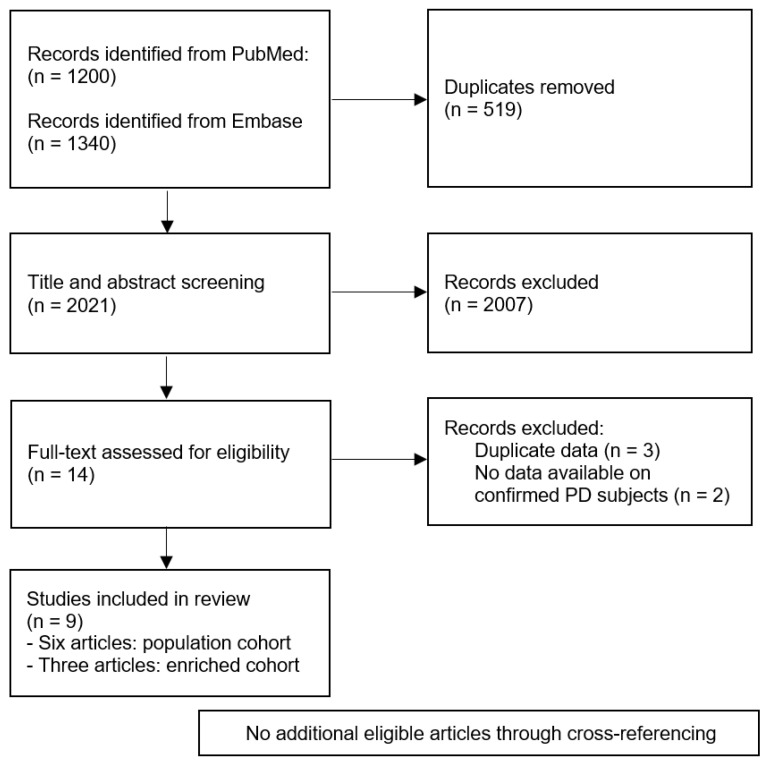
PRISMA flow diagram for the article selection process.

A total of 2021 unique articles were retrieved in the original search. Out of these articles, fourteen met the inclusion criteria before checking for overlapping data [18,19,20,21,23,25,26,27,28,29,30,31,32,33]. Three articles contained overlapping data, and as a result, the articles that were smaller or presented less data were excluded [30,31,32]. Moreover, two observational cohort studies that assessed cognition at baseline but did not present separate estimates for incident PD strata were excluded [28,29]. This left a total of nine articles for the analyses, which covered 215 individuals with manifest PD at follow-up and 13,524 individuals remaining disease-free. This process is displayed in Figure 1 and Figure 2, and characteristics are presented in Table 1.

Six articles reported data from a population cohort [19,20,21,23,26,33]. The other three articles reported data from an enriched cohort: two on individuals with idiopathic RBD [18,25] and one on individuals with hyposmia [27]. The average age in the studies ranged from 57.5 to 79.4 years. Mean or median duration of follow-up ranged from 2.6 to 15 years. Some studies used extensive batteries of cognitive tests and published the combined score [20,23,27], whereas others published the results of each separate task [18,19,20,21,23,25,26,33]. Results per domain are presented in Table S2.

### 3.2. Global Cognition: Screening Tests

All but one [33] of the included articles investigated global cognition (13,171 individuals included), either through a combined score of selected cognitive tests (author’s discretion) or through validated screening tests such as (versions of) the Mini-Mental State Examination (MMSE) and Montreal Cognitive Assessment (MoCA)

**Figure 3:**
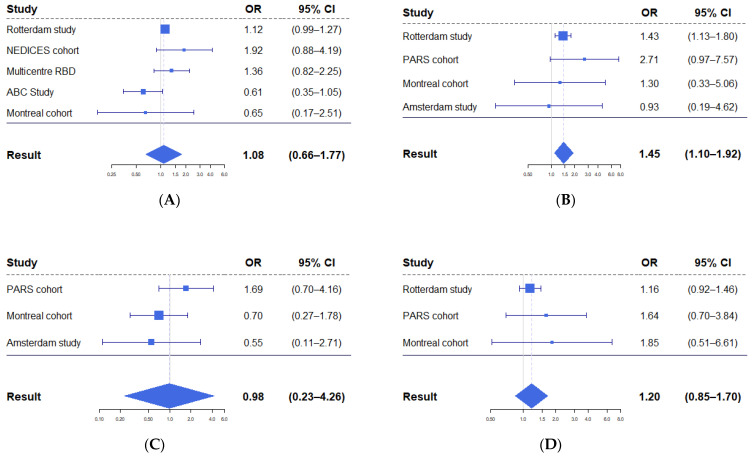
Forest plots displaying results from meta-analyses of odds of conversion to PD per standard deviation of worse performance. (**A**) Meta-analysis of global cognition scores. (**B**) Meta-analysis of executive function scores. In both the Rotterdam and Montreal cohorts, multiple tests were used to assess executive functioning. (**C**) Meta-analysis of attention scores. (**D)** Meta-analysis of learning and memory scores.

Five studies employed data suitable for meta-analysis on screening tests (MoCA or versions of the MMSE) for incident PD, varying between 0.61 and 1.92, with a pooled estimate of 1.08 (95% CI 0.66–1.77, Figure 3A).

### 3.3. Global Cognition: Combined Score

Three studies (7990 individuals included) [20,23,27] presented data on global cognition in combined scores with odds ratios varying between 1.09 and 2.55. Two out of three presented statistically significant outcomes; however, because of noncomparable risk metrics, no meta-analysis could be performed.

### 3.4. Executive Function

Six studies (8220 individuals included) [18,20,21,23,27,33] assessed executive functions using one or more of the following tests: the Stroop Colour Word Test, category fluency, letter fluency, the letter–digit substitution test, the Vienna perseveration task, and the Trail Making Test part B. The odds ratio of worse executive function for incident PD varied between 0.91 and 2.71, with a pooled estimate of 1.45 (95% CI 1.10–1.92, Figure 3B).

### 3.5. Attention

Four studies (576 individuals included) [18,21,27,33] determined the attention domain using one or more of the following tests: the Trail Making Test part A, the Digit Span Forward test, the Digit Symbol Coding test, the Corsi block test, and the symbol search test. The odds ratios of worse attention performance for incident PD varied between 0.51 and 1.69, with a pooled estimate of 0.98 (95% CI 0.23–4.26, Figure 3C).

### 3.6. Learning and Memory

Five studies assessed (8077 individuals included) [18,20,21,23,27] learning and memory using one or more of the following tests: word learning (immediate and delayed recalls and recognition), the Benton Visual Retention Test, the Digit Span Test backward, the California Verbal Learning Test (CVLT), the Rey Auditory–Verbal Learning Test, and selected tests from the Repeatable Battery for the Assessment of Neuropsychological Status (RBANS) A battery. The odds ratios of worse learning and memory performance for incident PD varied between 0.85 and 1.90, with a pooled estimate of 1.20 (95% CI 0.85–1.70, Figure 3D).

### 3.7. Language

Three studies (644 individuals included) [20,21,27] assessed the language domain through the Boston Naming Test. The odds ratios for worse language performance for incident PD varied between 0.78 and 1.64.

### 3.8. Visuospatial Function

Four studies (691 individuals included) [18,20,21,27] assessed visuospatial function using one or more of the following tests: the card rotation test, the Rey–Osterrieth complex figure test, the Bells omissions test, the block design test, and constructional praxis, copying and recall. The odds ratios of worse visuospatial performance for incident PD varied between 0.63 and 1.42.

## 4. Discussion

In this systematic review, we show that worse cognitive performance in specific cognitive domains is associated with an increased risk of conversion to PD. This effect seems to be more pronounced for tests measuring executive function, but not for tests regarding the attention or learning and memory domains.

However, the present review also lays bare several remaining gaps in knowledge, which we discuss in the next paragraphs.

Published empirical data on global and domain-specific cognitive deficits in prodromal PD is scarce, possibly because of the relative novelty of the concept of prodromal PD combined with the amount of time it takes for prodromal studies to be designed and executed. Furthermore, published studies were highly heterogeneous in study design, study population, and cognitive test batteries. This heterogeneity may explain some of the discrepancies between studies. Specifically, studies that used extensive cognitive test batteries reported an association between cognitive performance and onset of manifest PD [20,23,27], while studies that used only cognitive screening measures (e.g., MMSE or MoCA) did not [19,20,21,23,25,26]. Furthermore, similar domain-specific cognitive deficits that we identified here in the prodromal phase of PD have also been observed in previous clinical studies on cognitive function in newly diagnosed PD patients [34,35,36]. Diminished global cognitive performance was found in multiple studies, and among the specific cognitive subdomains, executive function was consistently found to be affected, especially in category fluency [18,23]. Interestingly, worse executive function performance—particularly category fluency—also predicted later conversion to PD dementia [37,38]. Performance on attention and memory domain tests was also commonly affected, although worse executive function might have influenced those assessments. Based on clinical studies, there might be two patterns of cognitive dysfunction in newly diagnosed PD patients: one phenotype with frontal executive dysfunction, possibly based on disruption of dopaminergic frontostriatal pathways, and the other mainly dominated by impaired visual orientation due to nondopaminergic dysfunction and cortical Lewy body deposition [39]. An area for future study is to assess whether separate patterns indeed emerge during the prodromal phase of PD and beyond. In addition, future studies should aim to compare results between unselected general population studies and enriched cohorts, which may differ considerably based on the underlying mechanisms of cognitive deficits [40].

A review from 2017 included longitudinal cohort, case-control, cross-sectional, and retrospective studies and elegantly described the relation between impaired cognitive function and prodromal or early PD [41]. It concluded that global cognition, and in particular executive function, might be affected in the prodromal phase. Our study expanded upon this finding by including additional original, recent longitudinal studies and provided a quantification of the association between global and domain-specific cognitive deficits and risk of manifest PD. It was purposefully decided to include only longitudinal cohorts in our study to provide a realistic quantification, minimizing selection bias and, consequently, emulating real-life risk estimation.

The pooled estimates presented in this study should be interpreted with caution because of two key limitations. First, results were meta-analysed from studies that were heterogeneous in the assessment of cognitive domains and tests. Our rationale for still meta-analysing estimates based on heterogeneous estimates was that global cognition scores derived from different (diverse) cognitive test batteries are generally similar [42,43]. Furthermore, for domain-specific analyses, we allocated cognitive tests to domains in accordance with the previous literature [14,15,16,17]. In reality, no cognitive test assesses purely one cognitive domain, and some overlap is inevitable. For example, recent literature has suggested that category fluency might depict verbal abilities besides executive function, whereas letter fluency is linked more strongly to executive function [44]. Consequently, we cannot exclude the possibility that heterogeneity in cognitive assessments between studies may have introduced some information error in the meta-analyses. To reduce the likelihood of a type I error, we used the conservative additional Hartung–Knapp correction. Second, the meta-analyses were based on summary statistics rather than individual participant data, and there was substantial variability in which risk metrics were reported. This might in part explain the differences in effect size between studies. Specifically, most articles reported the mean and standard deviation of baseline cognitive performance stratified by incident PD status but did not report time-dependent estimates of associations between cognition and incident PD. Consequently, we could calculate only a single odds ratio per association, which inevitably reduced the comparability of the results derived from studies with different follow-up durations, since cognitive deficits emerge gradually during the prodromal phase of PD [23,45]. It seems plausible that other population characteristics, such as mean age, may also have modified the pooled risk estimates. Unfortunately, the limited number of articles eligible for inclusion in this study precluded meta-regression analyses. Because of these limitations, the estimates in our meta-analyses should be considered as an indication rather than a robust quantification of true (biological) associations.

It should be noted that it was not possible to conduct a comprehensive meta-analysis of affected cognitive domains because of the relatively limited amount of published literature in this emerging field. Specifically, we could not meta-analyse the results of the language and visuospatial domains, which are both implicated in clinically manifest PD, since fewer than three published studies reported comparable risk metrics for these cognitive domains. This limitation precluded a head-to-head comparison of domain-specific results.

Lastly, because of the small number of studies included, no additional analyses for publication bias such as a funnel plot were performed.

To build on this review, collaborative observational studies with adequate follow-up should be conducted. Ideally, these studies would investigate domain-specific cognitive deficits and present comparable risk statistics that could be included in quantitative analyses and subsequently be incorporated in the MDS criteria for prodromal PD or other similar risk-scoring systems. To facilitate implementation in clinical practice, and to additionally boost inclusion of participants in future preventive trials, studies should also report binary risk estimates based on cutoff values for cognitive performance.

Furthermore, to facilitate serial remote assessment of cognitive performance, participant-reported outcome measures of cognitive deficits in prodromal PD should be developed.

## Figures and Tables

**Table 1:** Overview of included studies.

Cohort Name	Cohort Design	N * at Risk	N * Incident PD	Mean Age, yrs **	% Females	Duration of Follow-Up, yrs (Range) **
Montréal cohort [18]	Enriched iRBD cohort	47	10	70.7	27.0	3.5 (1.0–6.0)
ABC Study [19]	Population-based cohort	2424	42	75.6	51.6	10
Homburg/Saar cohort [20]	Population-based cohort	468	5	57.5	53.0	6.7 (2.0–10.1)
BLSA cohort [21]	Population-based cohort	40	10	79.4	20.0	2.6 (1.0–5.3)
Rotterdam Study [23]	Population-based cohort	7386	57	65.3	57.4	8.3 (0.0–15.0)
NEDICES cohort [26]	Population-based cohort	2450	21	72.8	57.2	3.4 (2.9–3.9)
PARS cohort [27]	Enriched cohort (hyposmia/familial)	136	8	67.1	38.0	3.8 (1.0–5.0)
Multicentre iRBD [25]	Multicentre enriched iRBD cohort	430	57 ***	67.4	19.0	3.6
Amsterdam study [8]	Enriched cohort (familial)	353	5	58.8	56.7	5

* N is the number of individuals per group. ** The numbers presented are the median for the Rotterdam study and the means for all other studies. *** The numbers presented are in reference to all persons in the cohort converting to PD; cognitive tests were administered on a subgroup. It is expected that the numbers presented in the table resemble the actual numbers. No regression was performed, and as such, this was deemed an acceptable surrogate.

## Data Availability

All data used in this study is available through the main text and the Supplementary Materials.

## Supplementary Materials

The following supporting information can be downloaded at: https://www.mdpi.com/article/10.3390/brainsci12020199/s1, Table S1: Overview of cognitive tests and their respective domains, Table S2: All cognition outcomes by domain.
