# Supplementary material for: Prodromal Cognitive Deficits and the Risk of Subsequent Parkinson’s Disease
Source: Brain Sci. 2022 Jan 31;12(2):199. doi: 10.3390/brainsci12020199 (PMC8870093; doi:10.3390/brainsci12020199)
Supplement: Supplementary file 1 [file brainsci-12-00199-s001.zip › brainsci-1526544-supplementary.pdf]

Supplementary materials:

Search strategy

The following search string was used to gather the articles of interest in this systematic review in PubMed:

(Parkinson* OR Parkinson Disease[mesh] OR Parkinson's disease OR Parkinson) AND (Premotor* OR prodromal* OR Preclinical* Or Pre-clinical* OR Prediagnostic* OR pre-diagnostic OR conversion OR convert OR "at risk") AND (Cognit* OR executive function OR visuospatial* OR attention* OR memory* OR fluency* OR processing speed OR verbal learning)

In Embase:

(Parkinson* OR Parkinson Disease/ OR Parkinson's disease OR Parkinson) AND (Premotor* OR prodromal* OR Preclinical* Or Pre-clinical* OR Prediagnostic* OR pre-diagnostic OR conversion OR convert OR at risk) AND (Cognit* OR executive function OR visuospatial* OR attention* OR memory* OR fluency* OR processing speed OR verbal learning)

Table S1. Overview of cognitive tests and their respective domains

Cognitive domain	*Cognitive test employed*
Global cognition	- Mini-Mental State Examination (MMSE)<br>- 37-item MMSE<br>- Montreal Cognitive Assessment or MoCA<br>- Consortium to Establish a Registry for Alzheimer's Disease or CERAD<br>- modified Teng Mini-Mental State Examination (3MS)
Executive function	- Category fluency<br>- Letter fluency<br>- Stroop Color Word Test<br>- Letter-Digit substitution<br>- Digit Symbol substitution*<br>- Trail Making Test, part B<br>- Vienna perseveration task
Attention	- Digit Span Test Forward<br>- Digit Span Test Backward*<br>- Trail Making Test, part A<br>- Symbol search test<br>- Corsi block test**
Learning and memory	- Benton Visual Retention Test<br>- Word learning, immediate/delayed recall<br>- Word learning, recognition<br>- California Verbal Learning Test, immediate/short delay/long delay free recall

	- Rey Auditory-Verbal Learning Test: List B, immediate/delayed recalls, recognition<br>- Repeatable Battery for the Assessment of Neuropsychological Status (RBANS) A battery, immediate and delayed recall, figure recall
Language	- Boston Naming Test
Visuospatial function	- Rey-Osterrieth Complex Figure<br>- Clock Drawing Test<br>- Block Design subtest (Wechsler Adult Intelligence Scale)<br>- Bells Test (omissions)<br>- Card rotation test<br>- Constructional praxis copying/recall

** One study [ins ref Weintraub et al] presented combined scores, employing multiple cognitive tests per domain. Through the method described in the main text (section 2.3 Data analysis) we allocated the Digit symbol substitution test in the executive function as opposed to the cohort's inclusion in the attention domain[ins ref Ibrahim-Verbaas et al]. Similarly, we allocated the Digit span backward test into the attention domain as opposed to the cohort's allocation to the executive domain[ins ref Daffner et al] . It was assumed, given the multitude of other tests included in the combined score that were allocated in their respective domain, that the resulting combined score still largely depicts the actual performance in that domain despite overlap.*

***The Amsterdam study[ins ref Ponsen et al] included the Corsi block test in the executive domain, however the most recent perspective would be that it depicts the attention domain and it was interpreted as such.*

Table S2. All cognition outcomes by domain

Study	*Cognitive measure*	*Odds ratio of worse cognition for conversion to clinical PD (95% CI)*
Global cognition, screening		
BLSA cohort	MMSE	- *
ABC Study	Teng-MMSE	OR 0.61 (0.34-1.05)**
Rotterdam Study	MMSE	HR 1.12 (0.99-1.27)
NEDICES cohort	37-MMSE	OR 1.92 (0.88-4.19)[3]
Homburg/Saar cohort	MMSE	OR 1.59 (1.04-2.44)
Multicentre RBD	MoCA	OR 1.36 (0.82-2.25)[3]
Montreal cohort	MMSE	OR 0.65 (0.17-2.51)**
Global cognition, combined		
Rotterdam Study	Combined score[1]	HR 1.52 (1.11-2.08)
PARS cohort	Combined score[2]	OR 2.55 (0.92-7.09)
Homburg/Saar cohort	CERAD	OR 1.09 (1.01-1.16)
Executive function		
Rotterdam Study	Stroop Color Word Test	HR 1.56 (1.20-2.00)
	Letter-Digit substitution	HR 1.32 (0.96-1.79)
	Category fluency	HR 1.35 (0.99-1.82)
PARS cohort	Combined score[4]	OR 2.71 (0.97-7.57)
Homburg/Saar cohort	Category fluency	- *
BLSA cohort	Category fluency	- *
	Letter fluency	- *
	Digit Symbol substitution	- *
	TMT-B	- *
Montreal cohort	Category fluency	OR 2.45 (0.64-9.31)**
	Letter fluency	OR 1.00 (0.26-3.77)**
	TMT-B	OR 0.91 (0.24-3.41)**
Amsterdam study	Perseveration task	OR 0.93 (0.19-4.62)[3]
Attention		
PARS cohort	Combined score[5]	OR 1.69 (0.69-4.16)
BLSA cohort	Digit Span Test Forward	- *
	TMT-A	- *
Montreal cohort	TMT-A	OR 0.94 (0.25-3.54) **
	Digit Span test	OR 0.51 (0.14-1.95)**
Amsterdam study	Corsi block test	OR 0.55 (0.11-2.71)[3]
Learning and Memory		
Rotterdam Study	Word learning, immediate recall	HR 1.19 (0.88-1.59)
	Word learning, recognition	HR 1.22 (0.99-1.49)
	Word learning, delayed recall	HR 1.02 (0.77-1.37)
PARS cohort	Combined score[6]	OR 1.64 (0.70-3.84)

Homburg/Saar cohort	Word list, immediate recall	OR 1.54 (1.14-2.13)
	Word list, delayed recall	- *
	Word learning, discriminability	- *
BLSA cohort	CVLT[7], immediate recall	- *
	CVLT, short-delay recall	- *
	CVLT, long-delay recall	- *
	BVRT[8]	- *
	Digit Span Test Backward	- *
Montreal cohort	RAVLT[9], total	OR 1.85 (0.51-6.61)**
	RAVLT, list B	OR 0.85 (0.44-1.89)**
	RAVLT, immediate recall	OR 1.93 (0.51-7.29)**
	RAVLT, delayed recall	OR 1.90 (0.50-7.16)**
	RAVLT, recognition	OR 1.84 (0.48-6.92)**
Language		
PARS cohort	BNT[10]	OR 0.78 (0.29-2.10)
Homburg/Saar cohort	BNT	OR 1.64 (1.01-2.70)
BLSA cohort	BNT	-*
Visuospatial Function		
PARS cohort	Combined score[11]	OR 1.42 (0.68-2.96)
Homburg/Saar cohort	Constructional praxis, copying	-*
	Constructional praxis, recall	-*
BLSA cohort	Card Rotation test	-*
Montreal cohort	ROCF[12], copy	OR 0.81 (0.21-3.03)**
	Bells Test, omissions	OR 0.92 (0.24-3.45)**
	Block Design	OR 0.63 (0.17-2.39)**

** No odds ratios could be calculated*
*** Calculated from raw (z-score) data, provided by author*

1. *Combined score for the Rotterdam study included: Letter-Digit substitution, Stroop Color Word Test, Verbal fluency (category), and Word learning, delayed recall.*
2. *Combined score for the PARS cohort study included an unweighted mean of the z scores for each domain combined.*
3. *Calculated from presented outcomes*
4. *Combined scores for the PARS cohort study included an unweighted mean of the z scores for each cognitive test conducted, including: letter and category fluency, TMT-B, Digit Span Backwards.*
5. *Combined scores for the PARS cohort study included an unweighted mean of the z scores for each cognitive test conducted, including: Digit symbol coding test, symbol search test, digit span forward test and the TMT-A.*
6. *Combined scores for the PARS cohort study included an unweighted mean of the z scores for each cognitive test conducted, including: immediate and delayed recall and recognition, the RBANS*

battery A immediate/delayed story recall, and the WMS-III logical memory story A (immediate and delayed recall)
7. CVLT: California Verbal Learning Test
8. BVRT: Benton Visual Retention Test
9. RAVLT: Rey Auditory-Verbal Learning Test
10. BNT: Boston Naming Test
11. Combined scores for the PARS cohort study included an unweighted mean of the z scores for each cognitive test conducted, including: RBANS Battery A Figure copying and line orientation, clock drawing test, and Visual Object and Space Perception Battery.
12. ROCF: Rey-Osterrieth Complex Figure.
